# Network analysis of the relationships between problematic smartphone use and anxiety, and depression in a sample of Chinese college students

**DOI:** 10.3389/fpsyt.2023.1097301

**Published:** 2023-04-17

**Authors:** Zhihua Guo, Tianqi Yang, Rui Qiu, Huake Qiu, Lei Ren, Xufeng Liu, Zheyi Han, Xia Zhu

**Affiliations:** ^1^Department of Military Medical Psychology, Air Force Medical University, Xi'an, China; ^2^Department of Gastroenterology, Air Force Medical Center, Air Force Medical University, Beijing, China

**Keywords:** problematic smartphone use, anxiety, depression, bridge node, network analysis

## Abstract

**Background:**

Problematic smartphone use (PSU) is associated with both anxiety and depression. However, the relationships between components of PSU and symptoms of anxiety or depression have not been investigated. Hence, the aim of this study was to closely examine the relationships between PSU and anxiety and depression to identify the pathological mechanisms underpinning those relationships. A second aim was to identify important bridge nodes to identify potential targets for intervention.

**Methods:**

Symptom-level network structures of PSU and anxiety, and PSU and depression were constructed to investigate the connections between the variables and evaluate the bridge expected influence (BEI) of each node. Network analysis using data from 325 Chinese healthy college students was performed.

**Results:**

Five strongest edges appeared within the communities in both the PSU-anxiety and PSU-depression networks. The “Withdrawal” component had more connections with symptoms of anxiety or depression than any other PSU node. In particular, the edges between “Withdrawal” and “Restlessness” and between “Withdrawal” and “Concentration difficulties” were the strongest cross-community edges in the PSU-anxiety network and PSU-depression network, respectively. Furthermore, “Withdrawal” had the highest BEI in the PSU community in both networks.

**Conclusions:**

These findings provide preliminary evidence of the pathological pathways linking PSU with anxiety and depression, with “Withdrawal” linking PSU with both anxiety and depression. Hence, “Withdrawal” may be a potential target for preventing and intervening in cases of anxiety or depression.

## 1. Introduction

The prevalence and incidence of anxiety and depression have increased substantially worldwide. In particular, young adults are susceptible to anxiety and depressive disorders. More than 20% individuals met criteria of anxiety disorders by early adulthood and the incidence of anxiety rose up to 47.1% in college students (1, 2), while the prevalence of depression reached 25% among university undergraduate students (3). A study also reported that the estimated prevalence of any depressive or anxiety disorder was 13.0% for graduate students (4–11). Anxiety and depression are not only harmful to mental health such as increased risk of suicidal thoughts and attempts (5, 6), but also correlate with physical diseases (7), including Parkinson's disease and cardiovascular disease (8, 9). The high incidences of anxiety and depressive disorders, and disability associated with depression and anxiety make them leading causes of the global burden of disease (10). Before effective interventions can be developed, however, it is essential to identify the pathogenesis of anxiety and depression.

The pathological mechanisms underpinning anxiety and depression have been explored in numerous studies. For example, the history of depression and affective instability are predictors of depression (11, 12), while the maltreatment, extreme behavioral inhibition, and parental overprotection are risk factors of anxiety disorders (13–15, 23–28). Problematic smartphone use (PSU) is defined as “an inability to regulate one's use of the smartphone, which eventually involves negative consequences in daily life” (16); due to the increasing PSU in young adults worldwide and its close associations with various facets of mental health (17), it has received increasing attention in the study of the pathogenesis of anxiety and depression. A systematic review of prevention and intervention strategies for smartphone addiction in students: applicability during the COVID-19 pandemic (31). Previous studies have found that PSU may lead to an increased risk of developing anxiety with which it is positively associated (18–21). Not only does PSU directly influence anxiety but it also has an indirect influence through sleep disturbance and bedtime procrastination (18, 22). In addition, PSU is closely related to depression and has been found to be a predictor of developing depression (19, 21–24). As previous research has noted, depression is positively correlated with process motivation of smartphone use, and process motivation has both direct and indirect effects on PSU through actual smartphone use (25).

However, previous studies have tended to view PSU, anxiety, or depression as a whole when investigating the relationships between PSU and anxiety or depression. However, PSU, anxiety, and depression are multi-variable constructs composed of distinct symptoms. The commonly used sum score based on the notion of symptom equivalence ignores the heterogeneity of symptoms (26), while the importance of symptoms actually varies (27). For example, individuals with the same sum score may be considered to have the same degree of depression, however, some may have high scores on symptoms of anhedonia and low scores on symptoms of fatigue while others may demonstrate the reverse pattern. Thus, they should have different levels of depression according to the relative importance of anhedonia and fatigue. Moreover, the single summative score obscures the specific relationships between individual symptoms. Hence, investigations into the associations between PSU and anxiety and between PSU and depression at a fine-grained level are essential to understand the pathological pathways linking PSU to anxiety or depression. Improved understanding of these associations may identify appropriate targets for effectively curbing the impacts of PSU on anxiety and depression.

A promising statistical method allowing the fine-grained analysis of the relationships between different variables is network analysis. Based on graph theory, network analysis conceptualizes psychopathological constructs as a network of interconnecting nodes (psychopathological variables) and edges (associations between variables) (28). Network analysis is a suitable tool to explore the elaborate associations between PSU and anxiety and between PSU and depression. It overcomes the drawbacks of previous studies that have considered psychopathological variables to be passive reflections of the underlying latent constructs (29–31). Network analysis allows the complex associations between different variables to be visualized (29, 32, 33). It also provides bridge centrality indices to assess the relative importance of a given variable in bridging different communities within the network. The term “community” is used to indicate a theoretically based group of psychological variables rather than based on any methods of network analysis such as community detection (34). These identified variables are called bridge nodes, which are critical to maintaining the co-occurrence of mental disorders and facilitating the contagion of one disorder to another or the adverse effects of one disorder on another (35–37). However, to the best of our knowledge, no study has investigated the relationships between PSU and anxiety or depression *via* network analysis.

To address this research gap, we investigated the relationships between components of PSU and symptoms of both anxiety and depression using network analysis. We constructed two networks to explore the associations between PSU and anxiety communities and between PSU and depression communities, respectively. We aimed to examine the links between PSU and both anxiety and depression to identify the pathological mechanisms underpinning them, determine important bridge nodes, and identify promising targets for intervention. Improved understanding of the specific roles of different PSU components in the development and maintenance of anxiety and depression, is essential to identify possible targets for clinically therapeutic interventions. Overall, this investigation is largely novel and exploratory and aimed to provide a new perspective of the relationships between PSU and both anxiety and depression.

## 2. Methods

### 2.1. Ethical approval

This study was reviewed and approved by the Independent Ethics Committee of Tangdu Hospital of the Fourth Military Medical University. Electronic informed consent was obtained from each participant before commencing the study.

### 2.2. Participants

This study was conducted in the form of an online survey through Wenjuanxing platform (www.wjx.cn) from 27 April 2022 to 16 May 2022. A total of 343 participants were recruited *via* convenience sampling based on WeChat moments. The inclusion criteria were: (1) healthy adults (aged 18 years or above); and (2) college students (undergraduates, masters, or doctors) while participants were excluded if they reported a history of organic brain damage or mental disorder. Responses were considered invalid and excluded from the analyses if the survey was completed in < 100 s, indicating indiscriminate responding without careful consideration of each item. The final sample contained 325 participants. Participants were also informed that the data collection and analyses were anonymous and were asked to answer the questions honestly.

### 2.3. Measures

#### 2.3.1. Smartphone Application-Based Addiction Scale (SABAS)

The valid Chinese version of SABAS was used to assess the likelihood of PSU (38, 39). It comprises six items which are based on the six criteria of the addiction components model (salience, conflict, mood modification, tolerance, withdrawal, and relapse) (40, 41). For example, the item “If I cannot use or access my smartphone when I feel like, I feel sad, moody, or irritable” represents “Withdrawal”. All items are rated using a 6-point Likert type scale ranging from 1 = *strongly disagree* to 6 = *strongly agree*. Higher SABAS scores indicate a higher risk of developing PSU. The internal consistency of SABAS was fairly good in the present study (Cronbach's α = 0.83).

#### 2.3.2. Generalized Anxiety Disorder 7-Item Questionnaire (GAD-7)

The GAD-7 is a reliable self-report questionnaire used to assess the frequency of the most important diagnostic symptoms of GAD over the last 2 weeks (42). It comprises seven items, such as “Feeling nervous, anxious or on edge”, that are rated on a 4-point Likert type scale (0 = *not at all*, 1 = *several days*, 2 = *more than half the days*, and 3 = *nearly every day*). Higher GAD-7 scores suggest more severe symptoms of anxiety. The Cronbach's α coefficient of GAD-7 in the present study was 0.92, indicating the internal consistency was excellent.

#### 2.3.3. Patient Health Questionnaire-9 (PHQ-9)

The PHQ-9 is a widely used self-report questionnaire that evaluates the frequency of symptoms of depression over the last 2 weeks (43). It includes nine items, for example, “Thoughts that you would be better off dead or of hurting yourself in some way”. The questionnaire is rated using a 4-point Likert type scale (0 = *not at all*, 1 = *several days*, 2 = *more than half the days*, and 3 = *nearly every day*). The higher the total score, the higher the level of depression severity. The internal consistency of PHQ-9 in the present study was excellent (Cronbach's α = 0.92).

### 2.4. Statistical analysis

SPSS 22.0 software was used to conduct the descriptive statistics and calculate Cronbach's α coefficients of SABAS, GAD-7, and PHQ-9. RStudio 4.1.1 software was used for network construction and bridge centrality evaluation.

#### 2.4.1. Network construction

The *qgraph* package was used for Gaussian Graphical Models (GGM) construction to describe the correlations among items in PSU-anxiety network and PSU-depression network (28, 44). In the networks, red edges represent negative partial correlations while blue edges represent positive partial correlations, and wider and more saturated edges represent stronger partial correlations (45). The partial correlation between two nodes was estimated after statistical controlling for the other nodes in the network (45). In the present study, nodes were divided into different communities according to the psychological variables to which they belonged, namely PSU community, anxiety community, and depression community. The combination of the least absolute shrinkage and selection operator (LASSO) regularization with the Extended Bayesian Information Criterion (EBIC) was used to limit the number of spurious edges (28, 46, 47). Consistent with guidelines (28), we set the hyperparameter γ of the EBIC to 0.5. We adopted Spearman's correlation method to estimate the network structure because of the ordinal nature of the items. The Fruchterman-Reingold algorithm was used to lay out the network, and nodes with stronger correlations were placed closer together (28, 48).

The *bootnet* package was utilized to estimate the accuracy of the edge weights (28) and estimated 95% confidence intervals (CI) were calculated by non-parametric bootstrapping (1,000 bootstrapped samples). A narrower 95% CI represented more accurate edge weights and a more reliable network (49, 50). We conducted bootstrapping (1,000 bootstrapped samples) using the *bootnet* package to test whether there were significant differences between the edge weights of different node pairs (28).

#### 2.4.2. Bridge centrality evaluation

The *networktools* package was used to evaluate bridge centrality (37). In present study, we assessed bridge expected influence (BEI) of each node. BEI of a node is defined as the sum of the edge weights between this node with all nodes from other communities. A higher BEI value suggests greater relevance with other communities (37, 51).

The *bootnet* package was used for the stability test of BEI and testing whether BEI differences were significant (28). We conducted case-dropping bootstrapping (1,000 bootstrapped samples) to test the stability of BEI and used the correlation stability (CS) coefficient to quantitatively describe the stability. A CS coefficient larger than 0.25 indicates acceptable stability (28). In addition, we conducted bootstrapping (1,000 bootstrapped samples) to test the differences of the BEI indices of different nodes.

## 3. Results

### 3.1. Descriptive statistics

The mean age of 325 participants was 21.49 ± 3.73 years (mean ± SD, range = 18–36 years). All participants had received a college education or above and more than half of the participants were female (female: *n* = 178, 54.8%; male: *n* = 147, 45.2%). Participants reported the average time spent on using a smartphone per day was 6.62 ± 3.59 hours (mean ± SD). The abbreviation, mean scores, and standard deviations for each variable of the PSU, anxiety, and depression communities are shown in Table 1.

**Table 1:** Abbreviations, mean scores, and standard deviations for the study variables.

**Variables**	**Abb**	**M**	**SD**
**PSU components (SABAS)**			
Salience	PSU1	3.73	1.31
Conflict	PSU2	2.77	1.35
Mood modification	PSU3	3.79	1.29
Tolerance	PSU4	3.43	1.23
Withdrawal	PSU5	3.00	1.29
Relapse	PSU6	3.10	1.22
**Anxiety symptoms (GAD-7)**			
Nervousness or anxiety	A1	0.96	0.72
Uncontrollable worry	A2	0.81	0.79
Worry too much	A3	0.86	0.82
Trouble relaxing	A4	0.87	0.82
Restlessness	A5	0.54	0.72
Irritable	A6	0.75	0.73
Afraid something will happen	A7	0.51	0.72
**Depression symptoms (PHQ-9)**			
Anhedonia	D1	0.83	0.79
Depressed or sad mood	D2	0.73	0.74
Sleep difficulties	D3	0.84	0.86
Fatigue	D4	0.89	0.82
Appetite changes	D5	0.70	0.80
Feeling of worthlessness	D6	0.65	0.79
Concentration difficulties	D7	0.66	0.76
Psychomotor agitation/retardation	D8	0.48	0.71
Thoughts of death	D9	0.38	0.68

Abb, abbreviation; M, mean; SD, standard deviation; PSU, problematic smartphone use; SABAS, Smartphone Application-Based Addiction Scale; GAD-7, Generalized Anxiety Disorder 7-Item Questionnaire; PHQ-9, Patient Health Questionnaire-9.

### 3.2. Network analysis

#### 3.2.1. The PSU-anxiety network

**Figure 1:**
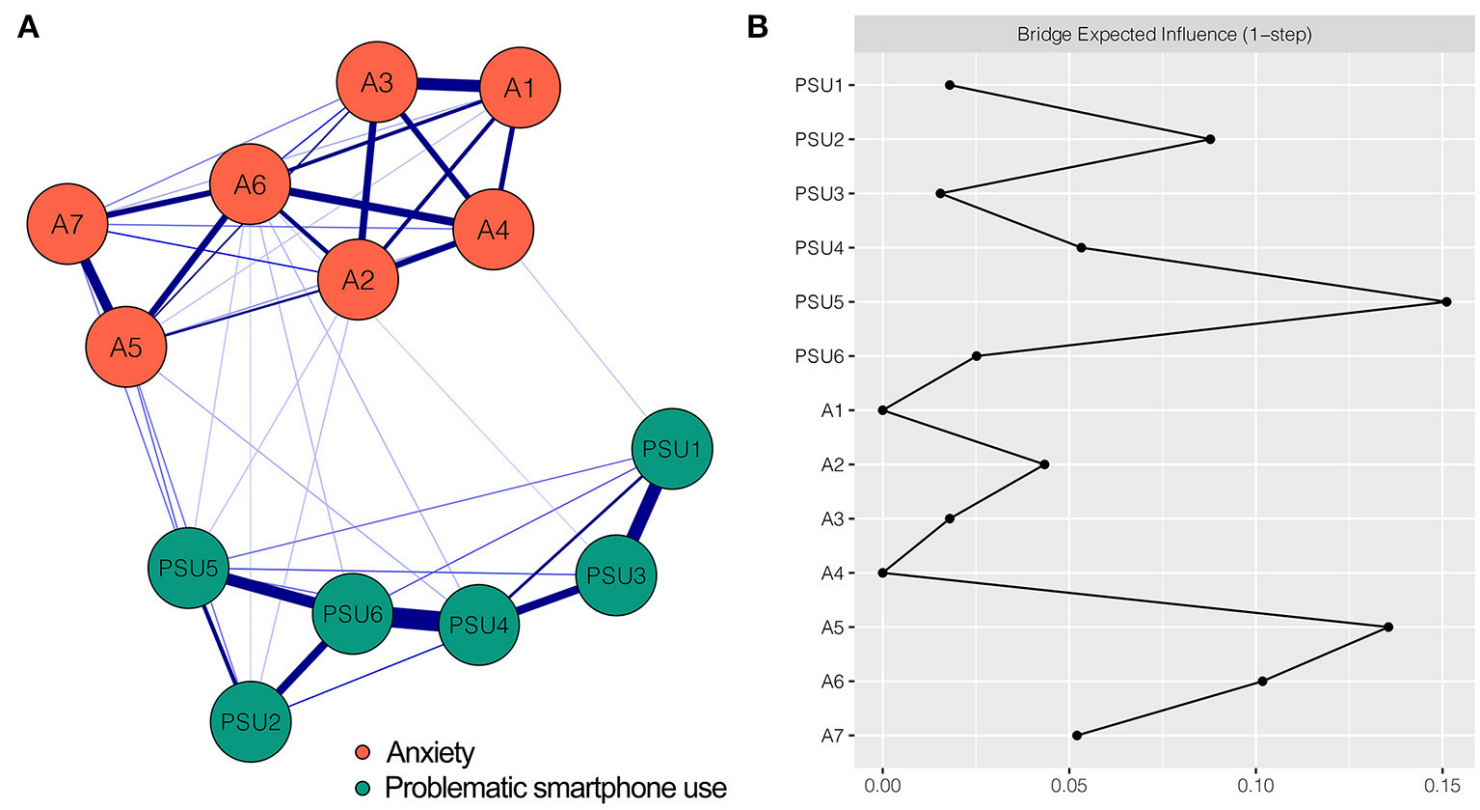
Network structure of PSU-anxiety variables and bridge expected influence for each node. **(A)** The PSU-anxiety network. Blue edges represent positive correlations. A thicker edge reflects higher correlation between the nodes. **(B)** The bridge expected influence of each node in the network (raw value). PSU1, Salience; PSU2, Conflict; PSU3, Mood modification; PSU4, Tolerance; PSU5, Withdrawal; PSU6, Relapse; A1, Nervousness or anxiety; A2, Uncontrollable worry; A3, Worry too much; A4, Trouble relaxing; A5, Restlessness; A6, Irritable; A7, Afraid something will happen.

The PSU-anxiety network is shown in Figure 1A, and has several important characteristics. First, 46 (59.0%) of 78 possible edges were non-zero and all edges were positive (weights ranging from < 0.01 to 0.45). Second, five strongest edges were identified in the final network. Three of the strongest edges were within the PSU community, namely the edges between PSU4 “Tolerance” and PSU6 “Relapse” (weight = 0.45), between PSU1 “Salience” and PSU3 “Mood modification” (weight = 0.33), and between PSU5 “Withdrawal” and PSU6 “Relapse” (weight = 0.29). Within the anxiety community, the two strongest edges were between A1 “Nervousness or anxiety” and A3 “Worry too much” (weight = 0.30) and between A5 “Restlessness” and A7 “Afraid something will happen” (weight = 0.26). Third, twelve cross-community edges were found in the network, although they were weaker than the within-community edges. We found PSU5 “Withdrawal” had more connections with symptoms of anxiety than other components of PSU. PSU5 “Withdrawal” was associated with A2 “Uncontrollable worry”, A5 “Restlessness”, A6 “Irritable”, and A7 “Afraid something will happen” (weight = 0.02, 0.06, 0.02, and 0.05, respectively). Furthermore, the strength of the edge between PSU5 “Withdrawal” and A5 “Restlessness” (weight = 0.06) was larger than any other cross-community edge. Supplementary Table 1 demonstrates all the edge weights within the PSU-anxiety network. Bootstrapped 95% CIs were narrow, indicating that the estimation of edge weights was accurate and reliable (see Supplementary Figure 1). The bootstrapped difference test for the edge weights revealed that the weights of the five strongest edges were significantly higher than 57.8% to 97.8% of the other edge weights (see Supplementary Figure 2).

The BEI of each node is shown in Figure 1B. Nodes PSU5 “Withdrawal” (BEI = 0.15) and A5 “Restlessness” (BEI = 0.14) exhibited highest BEIs. The BEI of PSU5 “Withdrawal” was the highest in the PSU community, emphasizing the impact of PSU5 “Withdrawal” on anxiety. The CS coefficient of node BEI was 0.28, exceeding the recommended threshold of 0.25, indicating that the BEI estimation was acceptable (see Supplementary Figure 3). The bootstrapped difference test for node BEI is shown in Supplementary Figure 4.

#### 3.2.2. The PSU-depression network

**Figure 2:**
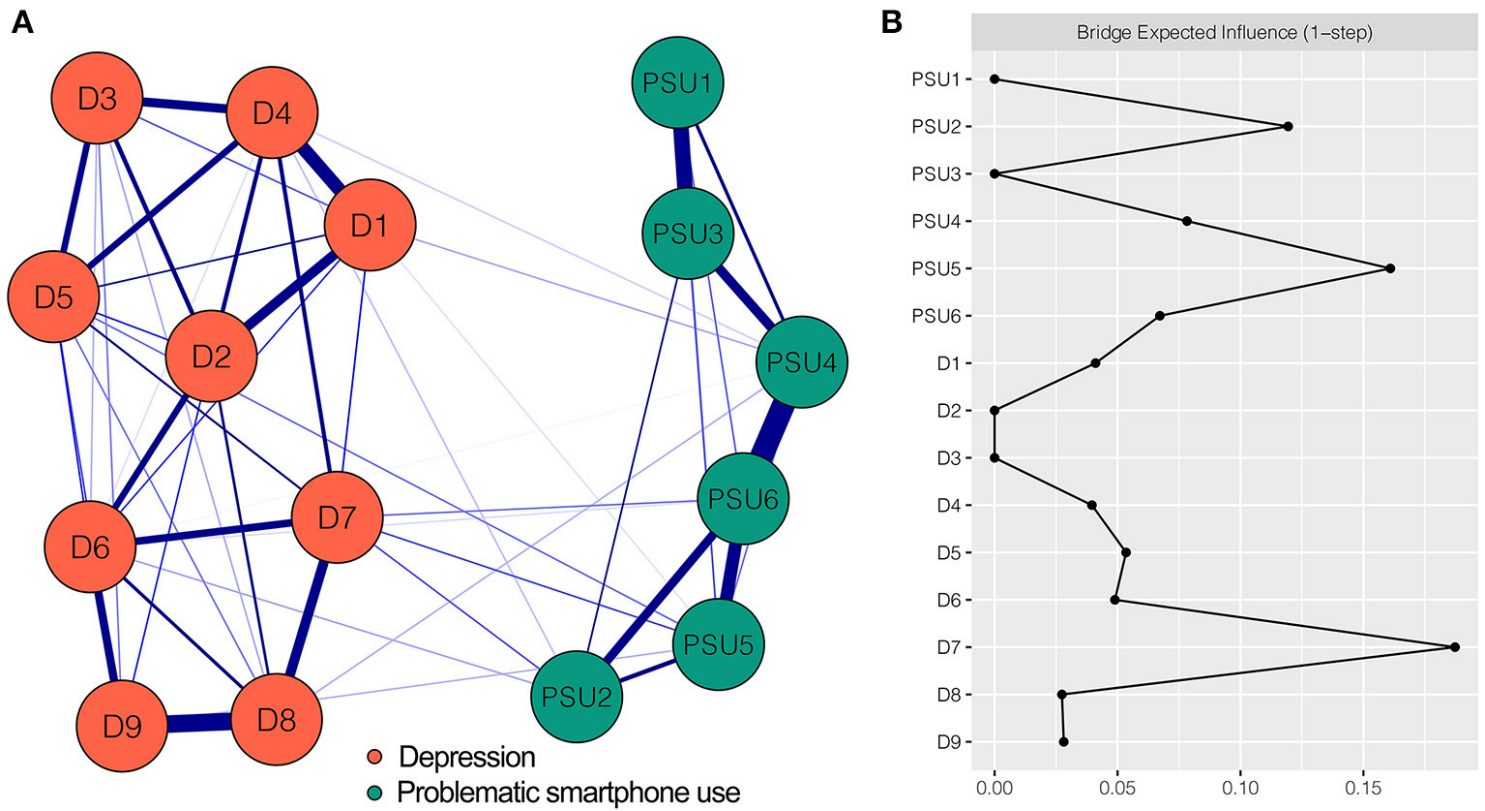
Network structure of PSU-depression variables and bridge expected influence for each node. **(A)** The PSU-depression network. Blue edges represent positive correlations. A thicker edge reflects higher correlation between the nodes. **(B)** The bridge expected influence of each node in the network (raw value). PSU1, Salience; PSU2, Conflict; PSU3, Mood modification; PSU4, Tolerance; PSU5, Withdrawal; PSU6, Relapse; D1, Anhedonia; D2, Depressed or sad mood; D3, Sleep difficulties; D4, Fatigue; D5, Appetite changes; D6, Feeling of worthlessness; D7, Concentration difficulties; D8, Psychomotor agitation/retardation; D9, Thoughts of death.

Figure 2A shows the PSU-depression network which has some noteworthy characteristics. First, there were 54 (51.4%) non-zero edges among 105 possible edges in this network with 15 nodes. All the edges were positive (weights ranging from < 0.01 to 0.45). Second, of the five identified strongest edges in the final network, three were within the PSU community, namely the edges between PSU4 “Tolerance” and PSU6 “Relapse” (weight = 0.45), between PSU1 “Salience” and PSU3 “Mood modification” (weight = 0.33), and between PSU5 “Withdrawal” and PSU6 “Relapse” (weight = 0.29). The other two strongest edges existed within the depression community, which were between D8 “Psychomotor agitation/retardation” and D9 “Thoughts of death” (weight = 0.36) and between D1 “Anhedonia” and D4 “Fatigue” (weight = 0.30). Third, thirteen connections between PSU and depression were revealed (i.e., cross-community edges). PSU5 “Withdrawal” had more connections with depression than other PSU components. It correlated with four depression symptoms: D7 “Concentration difficulties” (weight = 0.07), D5 “Appetite changes” (weight = 0.05), D9 “Thoughts of death” (weight = 0.03), and D1 “Anhedonia” (weight = 0.01). The strength of the edge between PSU5 “Withdrawal” and D7 “Concentration difficulties” (weight = 0.07) was larger than that of any other cross-community edge. All edge weights of the PSU-depression network can be seen in Supplementary Table 2. The bootstrapped 95% CIs for the estimated edge weights were relatively narrow, indicating the estimates were reliable (see Supplementary Figure 5). Result of the bootstrapped difference test for edge weights is shown in Supplementary Figure 6, revealing that the weights of the five strongest edges were significantly higher than 69.8–96.2% of the weights of other edges.

The BEI for each network node is shown in Figure 2B. Two nodes exhibited the highest BEIs. One was D7 “Concentration difficulties” (BEI = 0.19) in the depression community and the other was PSU5 “Withdrawal” (BEI = 0.16) in the PSU community. From the perspective of our research goals, PSU5 “Withdrawal” was more important than D7 “Concentration difficulties” insofar as the former had the highest BEI among the nodes of PSU community, suggesting that the bridge node PSU5 “Withdrawal” had enormous influence on depression. The CS coefficient for BEI was 0.44, exceeding the recommended threshold of 0.25, which indicated the estimation of BEI had an acceptable level of stability (see Supplementary Figure 7). The bootstrapped difference test for node BEI showed that the BEI of PSU5 “Withdrawal” was significantly higher than 50% of the other node BEIs in the current network (see Supplementary Figure 8).

Although it is beyond the scope of this study, the relevant results of the three-community network comprised of PSU, anxiety, and depression are provided in the Results in the Supplementary material, helping readers to access more information.

## 4. Discussion

The present study examined the network models of interactions between PSU and anxiety and depression. Overall, network analysis revealed within-community and cross-community edges and identified important bridge nodes which exerted a great deal of influence on anxiety and depression. Furthermore, both the PSU-anxiety network and PSU-depression network had acceptable robustness. To our knowledge, this is the first study to closely investigate the fine-grained relationships between PSU and anxiety and depression to better understand the pathological pathways linking PSU with anxiety and depression. We also identified critical bridge nodes that provide insights into potential targets for intervention and treatments for anxiety and depression.

The strongest edges in the PSU-anxiety network and PSU-depression network all appeared within the community rather than connecting different communities. This is consistent with many previous studies which examined the co-occurrence of different psychopathological constructs and found the strongest edges appeared within the community (36, 52–57). The three strongest edges within the PSU community in both networks were the same, which were between PSU4 “Tolerance” and PSU6 “Relapse”, between PSU5 “Withdrawal” and PSU6 “Relapse”, and between PSU1 “Salience” and PSU3 “Mood modification”. These findings are in line with previous studies that have used network analysis (52, 55). Within the anxiety community, the two strongest edges were between A1 “Nervousness or anxiety” and A3 “Worry too much” and between A5 “Restlessness” and A7 “Afraid something will happen”. This result is consistent with previous studies (53, 54). Within the depression community, the two strongest edges existed between D8 “Psychomotor agitation/retardation” and D9 “Thoughts of death” and between D1 “Anhedonia” and D4 “Fatigue”, which accords with the findings of a previous study (53). Together, these findings were expected because the closely associated variables were sub-components of a self-reported scale, which were either conceptually related or symptom related.

In addition to within-community edges, we found some edges connecting PSU and anxiety, and PSU and depression. These findings provide insights into the complex relationships between them. Consistent with previous studies that have shown that PSU is a risk factor for developing anxiety and depression (18, 19, 21–23), the present study further advances our understanding of the pathological pathways between PSU and anxiety and depression from the perspective of network structure. Of most interest was the finding that PSU5 “Withdrawal” had more connections with anxiety and depression than any other PSU component, indicating that individuals with the PSU “Withdrawal” component may be predisposed to anxiety and depression. The result is consistent with reports in previous studies that people feel unease, including anxiety and depression, when unable to use their smartphone or after abstinence (58, 59). Specifically, the strongest cross-community edge in the PSU-anxiety network was between PSU5 “Withdrawal” and A5 “Restlessness”, while the strongest cross-community edge was between PSU5 “Withdrawal” and D7 “Concentration difficulties” in the PSU-depression network. It indicated that individuals with PSU “Withdrawal” symptom are more liable to develop “Restlessness” symptom of anxiety and “Concentration difficulties” that are symptomatic of depression. This finding may underlie the pathological mechanisms which link PSU with anxiety and depression. Hence, the PSU “Withdrawal” component can be used to identify individuals at risk of developing anxiety or depression. However, since no studies have investigated the interrelations at such a detailed level, our study provides preliminary evidence of the possible mechanisms that need to be investigated in the future.

In the networks presented in this study, bridge nodes provide a new perspective of the co-occurrence of PSU and anxiety, as well as PSU and depression, and shed light on the specific roles played by different PSU components in the development and maintenance of anxiety and depression. Considering the theoretical and practical implications of bridge nodes, they are promising targets for intervention and treatment (36, 37, 52, 60). In the PSU-anxiety network, the node PSU5 “Withdrawal” was identified as the most important bridge node. This suggests that “Withdrawal” had stronger associations with symptoms of anxiety than other PSU components, thus exerting an important impact on anxiety and contributing to its development and maintenance. Therefore, the “Withdrawal” component may be a promising target for the prevention and treatment of anxiety, and may be more effective than targeting other PSU components. Similarly, in the PSU-depression network, the node PSU5 “Withdrawal” was determined to be the most important bridge node of all the PSU components. Consequently, it is recommended that “Withdrawal” be targeted in interventions for depression. As discussed earlier, individuals with “Withdrawal” symptom are susceptible to developing anxiety and depression, which also indirectly indicates a potential target for intervening.

Although the present study provides preliminary insights into the pathological pathways linking PSU with anxiety and depression, and presents a potential target for effectively intervening in cases of anxiety and depression, there are some limitations that warrant consideration. First, the cross-sectional design of this study precludes the examination of causal relationships or changes between variables over time. Longitudinal investigations of the relationships between PSU and anxiety or depression using network analysis are needed. Second, although we identified “Withdrawal” as a potential target for treatment, future prospective or longitudinal studies should examine whether interventions targeting “Withdrawal” component are effective. Third, PSU, anxiety, and depression were all assessed *via* self-report scales, that may be subject to recall bias and social approval effects (50, 59, 61), which means our findings must be interpreted cautiously. Fourth, given that the study utilized convenience sampling and recruited specific sample of healthy adults with high levels of education, it is not known how generalizable our findings are to other populations. Replication of these findings in other populations (e.g., clinical samples) is required to confirm their applicability to other groups. Fifth, the networks constructed in this study examined between-subject effects at a group level, and the network structure of a single individual may not be identical. Sixth, although a sample size of 325 is usually not considered as a small sample in experimental study, in network analysis field in which this study belongs our sample size is too small to be sufficiently representative of college students, so we only provided preliminary and exploratory findings. The results of this study can be further verified by expanding the sample size in the future. Finally, the study included only one scale to measure each construct that may not have captured all aspects of the three constructs. Therefore, future studies using additional scales that measure other aspects of PSU, anxiety and depression are recommended to more comprehensively investigate these relationships.

## 5. Conclusion

The present study is the first to simultaneously investigate the relationships between PSU and both anxiety and depression using network analysis. The results indicated that the PSU component “Withdrawal” was associated with symptoms of both anxiety and depression. By highlighting the cross-community edges between “Withdrawal” and “Restlessness” and between “Withdrawal” and “Concentration difficulties”, our study provides a fine-grained understanding of the pathological pathways linking PSU with anxiety and depression. The “Withdrawal” component was also identified as the critical bridge node, indicating that it plays an important role in the development and maintenance of anxiety and depression. Therefore, “Withdrawal” may be used to identify individuals at risk of developing anxiety or depression, and may be a potential target for the development of effective prevention and intervention strategies.

## Data availability statement

The raw data supporting the conclusions of this article will be made available by the authors, without undue reservation.

## Ethics statement

The studies involving human participants were reviewed and approved by the Ethics Committee of Tangdu Hospital of the Fourth Military Medical University. The patients/participants provided their written informed consent to participate in this study.

## Author contributions

Study concept and design: ZG, ZH, and XZ. Data collection, analysis and interpretation: ZG, TY, RQ, HQ, and LR. Writing–original draft: ZG and TY. Writing– revision and edit: XL, ZH, and XZ. Funding: XZ. All of the authors have approved the publication of this manuscript.
