# Supplementary material for: Network analysis of the relationships between problematic smartphone use and anxiety, and depression in a sample of Chinese college students
Source: Front Psychiatry. 2023 Apr 17;14:1097301. doi: 10.3389/fpsyt.2023.1097301 (PMC10149733; doi:10.3389/fpsyt.2023.1097301)
Supplement: Supplementary file 1 [file Data_Sheet_1.DOCX]

Supplementary Material

Supplementary Table 1. All edges weights within the PSU-anxiety network

|  | PSU1 | PSU2 | PSU3 | PSU4 | PSU5 | PSU6 | A1 | A2 | A3 | A4 | A5 | A6 | A7 |
| --- | --- | --- | --- | --- | --- | --- | --- | --- | --- | --- | --- | --- | --- |
| PSU1 | 0 |  |  |  |  |  |  |  |  |  |  |  |  |
| PSU2 | 0 | 0 |  |  |  |  |  |  |  |  |  |  |  |
| PSU3 | 0.33 | 0.10 | 0 |  |  |  |  |  |  |  |  |  |  |
| PSU4 | 0.13 | 0.0008 | 0.23 | 0 |  |  |  |  |  |  |  |  |  |
| PSU5 | 0.05 | 0.15 | 0.07 | 0.06 | 0 |  |  |  |  |  |  |  |  |
| PSU6 | 0.06 | 0.23 | 0 | 0.45 | 0.29 | 0 |  |  |  |  |  |  |  |
| A1 | 0 | 0 | 0 | 0 | 0 | 0 | 0 |  |  |  |  |  |  |
| A2 | 0 | 0.02 | 0 | 0 | 0.02 | 0 | 0.15 | 0 |  |  |  |  |  |
| A3 | 0.02 | 0 | 0 | 0 | 0 | 0 | 0.30 | 0.21 | 0 |  |  |  |  |
| A4 | 0 | 0 | 0 | 0 | 0 | 0 | 0.17 | 0.19 | 0.19 | 0 |  |  |  |
| A5 | 0 | 0.05 | 0 | 0.03 | 0.06 | 0 | 0.02 | 0.12 | 0.10 | 0.04 | 0 |  |  |
| A6 | 0 | 0.01 | 0.02 | 0.03 | 0.02 | 0.03 | 0.14 | 0.15 | 0.09 | 0.23 | 0.20 | 0 |  |
| A7 | 0 | 0 | 0 | 0 | 0.05 | 0 | 0.03 | 0.10 | 0.05 | 0.05 | 0.26 | 0.18 | 0 |

Supplementary Figure 1. Accuracy of edge weights in the PSU-anxiety network

*Note*: The red line depicts the sample edge weights and the gray bar depicts the bootstrapped confidence interval.

Supplementary Figure 2. Bootstrapped difference test for edge weights in the PSU-anxiety network

*Note*: Gray boxes indicate edge weights that do not differ significantly from one another, while black boxes indicate edge weights that do differ significantly. Blue boxes on the diagonal correspond to edge weights with positive correlations.

Supplementary Figure 3. Stability of node bridge expected influences in the PSU-anxiety network

*Note*: The red bar represents the average correlation between node bridge expected influences in the full sample and subsample with the red area depicting the 2.5th quantile to the 97.5th quantile.

Supplementary Figure 4. Bootstrapped difference test for node bridge expected influences in the PSU-anxiety network

*Note*: Gray boxes indicate node bridge expected influences that do not differ significantly from one another, while black boxes indicate node bridge expected influences that do differ significantly.

Supplementary Table 2. All edges weights within the PSU-depression network

|  | PSU1 | PSU2 | PSU3 | PSU4 | PSU5 | PSU6 | D1 | D2 | D3 | D4 | D5 | D6 | D7 | D8 | D9 |
| --- | --- | --- | --- | --- | --- | --- | --- | --- | --- | --- | --- | --- | --- | --- | --- |
| PSU1 | 0 |  |  |  |  |  |  |  |  |  |  |  |  |  |  |
| PSU2 | 0 | 0 |  |  |  |  |  |  |  |  |  |  |  |  |  |
| PSU3 | 0.33 | 0.10 | 0 |  |  |  |  |  |  |  |  |  |  |  |  |
| PSU4 | 0.13 | 0 | 0.23 | 0 |  |  |  |  |  |  |  |  |  |  |  |
| PSU5 | 0.06 | 0.15 | 0.07 | 0.05 | 0 |  |  |  |  |  |  |  |  |  |  |
| PSU6 | 0.06 | 0.22 | 0 | 0.45 | 0.29 | 0 |  |  |  |  |  |  |  |  |  |
| D1 | 0 | 0 | 0 | 0.03 | 0.01 | 0 | 0 |  |  |  |  |  |  |  |  |
| D2 | 0 | 0 | 0 | 0 | 0 | 0 | 0.24 | 0 |  |  |  |  |  |  |  |
| D3 | 0 | 0 | 0 | 0 | 0 | 0 | 0.06 | 0.15 | 0 |  |  |  |  |  |  |
| D4 | 0 | 0.02 | 0 | 0.02 | 0 | 0 | 0.30 | 0.14 | 0.22 | 0 |  |  |  |  |  |
| D5 | 0 | 0 | 0 | 0 | 0.05 | 0 | 0.10 | 0.08 | 0.18 | 0.18 | 0 |  |  |  |  |
| D6 | 0 | 0.03 | 0 | 0.003 | 0 | 0.01 | 0.08 | 0.18 | 0.03 | 0.01 | 0.09 | 0 |  |  |  |
| D7 | 0 | 0.06 | 0 | 0 | 0.07 | 0.05 | 0.10 | 0 | 0 | 0.14 | 0.11 | 0.20 | 0 |  |  |
| D8 | 0 | 0 | 0 | 0.03 | 0 | 0 | 0 | 0.12 | 0.03 | 0 | 0.05 | 0.13 | 0.24 | 0 |  |
| D9 | 0 | 0 | 0 | 0 | 0.03 | 0 | 0 | 0.08 | 0.05 | 0 | 0.08 | 0.21 | 0 | 0.36 | 0 |

Supplementary Figure 5. Accuracy of edge weights in the PSU-depression network

*Note*: The red line depicts the sample edge weights and the gray bar depicts the bootstrapped confidence interval.

Supplementary Figure 6. Bootstrapped difference test for edge weights in the PSU-depression network

*Note*: Gray boxes indicate edge weights that do not differ significantly from one another, while black boxes indicate edge weights that do differ significantly. Blue boxes on the diagonal correspond to edge weights with positive correlations.

Supplementary Figure 7. Stability of node bridge expected influences in the PSU-depression network

*Note*: The red bar represents the average correlation between node bridge expected influences in the full sample and subsample with the red area depicting the 2.5th quantile to the 97.5th quantile.

Supplementary Figure 8. Bootstrapped difference test for node bridge expected influences in the PSU-depression network

*Note*: Gray boxes indicate node bridge expected influences that do not differ significantly from one another, while black boxes indicate node bridge expected influences that do differ significantly.

Supplementary Results

The PSU-anxiety-depression network is shown in Figure 1. The bootstrapped 95% CIs for the estimated edge weights is shown in Figure 2. Figure 3 depicts the bootstrapped difference test for the edge weights. The expected influence (EI) of each node is shown in Figure 4. Nodes D2 “Depressed or sad mood” (EI = 1.13), A6 “Irritable” (EI = 1.14), and PSU6 “Relapse” (EI = 1.09) exhibited highest EIs. Figure 5 and 6 shows the stability and bootstrapped difference tests for node EI, respectively. The bridge expected influence (BEI) of each node is shown in Figure 7. Nodes D2 “Depressed or sad mood” (BEI = 0.52), A7 “Afraid something will happen” (BEI = 0.46), and A5 “Restlessness” (BEI = 0.45) exhibited highest BEIs. Figure 8 and 9 shows the stability and bootstrapped difference tests for node BEI, respectively.

Figure 1. Network structure of PSU, anxiety, and depression

Figure 2. Accuracy of edge weights in the PSU-anxiety-depression network

*Note*: The red line depicts the sample edge weights and the gray bar depicts the bootstrapped confidence interval.

Figure 3. Bootstrapped difference test for edge weights in the PSU-anxiety-depression network

*Note*: Gray boxes indicate edge weights that do not differ significantly from one another, while black boxes indicate edge weights that do differ significantly. Blue boxes on the diagonal correspond to edge weights with positive correlations.

Figure 4. The expected influence of each node in the PSU-anxiety-depression network (raw value)

Figure 5. Stability of node expected influences in the PSU-anxiety-depression network

*Note*: The red bar represents the average correlation between node expected influences in the full sample and subsample with the red area depicting the 2.5th quantile to the 97.5th quantile.

Figure 6. Bootstrapped difference test for node expected influences in the PSU-anxiety-depression network

*Note*: Gray boxes indicate node expected influences that do not differ significantly from one another, while black boxes indicate node expected influences that do differ significantly. The numbers in the white boxes (i.e., diagonal line) represent the values of node expected influences.

Figure 7. The bridge expected influence of each node in the PSU-anxiety-depression network (raw value)

Figure 8. Stability of node bridge expected influences in the PSU-anxiety-depression network

*Note*: The red bar represents the average correlation between node bridge expected influences in the full sample and subsample with the red area depicting the 2.5th quantile to the 97.5th quantile.

Figure 9. Bootstrapped difference test for node bridge expected influences in the PSU-anxiety-depression network

*Note*: Gray boxes indicate node bridge expected influences that do not differ significantly from one another, while black boxes indicate node bridge expected influences that do differ significantly.
